# The WHO Maternal Near Miss Approach: Consequences at Malawian District Level

**DOI:** 10.1371/journal.pone.0054805

**Published:** 2013-01-25

**Authors:** Thomas van den Akker, Jogchum Beltman, Joey Leyten, Beatrice Mwagomba, Tarek Meguid, Jelle Stekelenburg, Jos van Roosmalen

**Affiliations:** 1 Thyolo District Health Office, Ministry of Health, Thyolo, Malawi; 2 Department of Medical Humanities, EMGO Institute, VU University Medical Centre, Amsterdam, The Netherlands; 3 Working Party on International Safe Motherhood and Reproductive Health, Dutch Society of Obstetrics and Gynecology and the Netherlands Society of Tropical Medicine and International Health, Utrecht, The Netherlands; 4 Department of Obstetrics, Leiden University Medical Centre, Leiden, The Netherlands; 5 School of Law and Graduate School of Public Health, University of Pittsburgh, Pittsburgh, Pennsylvania, United States of America; 6 Department of Obstetrics and Gynecology, Bottom Hospital, Lilongwe, Malawi; 7 Department of Obstetrics and Gynecology, Leeuwarden Medical Centre, Leeuwarden, The Netherlands; Wits Reproductive Health and HIV Institute, South Africa

## Abstract

**Introduction:**

WHO proposes a set of *organ-failure* based criteria for maternal near miss. Our objective was to evaluate what implementation of these criteria would mean for the analysis of a cohort of 386 women in Thyolo District, Malawi, who sustained severe acute maternal morbidity according to *disease*-based criteria.

**Methods and Findings:**

A WHO Maternal Near Miss (MNM) Tool, created to compare disease-, intervention- and organ-failure based criteria for maternal near miss, was completed for each woman, based on a review of all available medical records. Using disease-based criteria developed for the local setting, 341 (88%) of the 386 women fulfilled the WHO disease-based criteria provided by the WHO MNM Tool, 179 (46%) fulfilled the intervention-based criteria, and only 85 (22%) the suggested organ-failure based criteria.

**Conclusions:**

In this low-resource setting, application of these organ-failure based criteria that require relatively sophisticated laboratory and clinical monitoring underestimates the occurrence of maternal near miss. Therefore, these criteria and the suggested WHO approach may not be suited to compare maternal near miss across all settings.

## Introduction

Maternal mortality remains one of the major public health problems of our time, and poor quality obstetric services continue to be an important associated factor [1]; [2] In order to identify and correct deficiencies in health care delivery, maternal mortality audits are performed in many health facilities throughout the world [3]. However, the absolute number of maternal deaths occurring at the level of health facilities is often low. Therefore, case reviews are increasingly directed at women who *survived* a serious health condition during pregnancy and childbirth, in addition to women who died [4]; [5]. Peer-review of severe morbidity has the added advantage of being potentially less threatening to the morale of participants compared to mortality audit: ‘near misses’ may sometimes be presented as ‘great saves’.

A pregnant or recently delivered woman who nearly died from a critical condition is often described as a ‘near miss’ or ‘severe acute maternal morbidity’. The WHO working group on maternal mortality and morbidity classifications (‘the working group’) proposed the term ‘maternal near miss’ (MNM) which can be defined as ‘any woman who nearly died but survived a complication that occurred during pregnancy, childbirth or within 42 days of termination of pregnancy’ [6].

In order to advance the use of the MNM concept and compare near miss estimates across settings and over time, the working group set out to arrive at uniform criteria for the identification of MNM. The choice is between three distinct types of criteria that have been used in the past: (A) clinical criteria related to a specific condition, such as eclampsia or hemorrhage (‘A-criteria’), (B) intervention-specific criteria such as admission into an intensive care unit or the performance of laparotomy or blood transfusion (‘B-criteria’), or (C) a set of criteria whereby organ system dysfunction such as shock or renal dysfunction is identified (‘C-criteria’) [6].

According to the working group, the ‘organ-system dysfunction based approach’ is the most promising of the three options and should form the basis for a standardized set of inclusion (type ‘C’) criteria. Since organ dysfunction may be regarded as a pre-stage to death, identifying organ failure in a woman suffering from pregnancy-related illness could be a way to identify those women at high risk of mortality. In this respect, the WHO working group makes a difference between potentially life-threatening conditions on the one hand (e.g. eclampsia or major hemorrhage) and life-threatening conditions on the other (those that have resulted in organ failure).

To ensure the usefulness of the organ-failure based approach in resource-poor settings, markers that can generally only be diagnosed by intensive care monitoring - which is usually not available in such settings - would need to be supplemented by simpler clinical markers such as gasping, oliguria or jaundice [6]. With the objective of evaluating the implications of an organ-system dysfunction based approach, WHO developed a ‘Maternal Near Miss Tool’ (WHO-MNM Tool) (Figure S1).

The objective of this study was to examine the implications of applying the WHO-MNM tool to a cohort of women who sustained severe maternal morbidity in an under-resourced district in sub-Saharan Africa, and, in this way, to assess the feasibility of the organ failure based approach in such a setting. In addition, we wanted to compare the feasibility and appropriateness of applying each of the three suggested types of MNM criteria (disease-, intervention- and organ-specific).

## Methods

### Ethical clearance

This study was a re-analysis of the same cohort that was published in PLoS ONE before [7], and it was performed in full accordance with the guidelines for operational research of the National Research Council and the Health Sciences Research Committee of the Ministry of Health of Malawi [8]; [9] and with the Helsinki declaration of 1975, as revised in 1983. For the original study, verbal approval had been obtained from the National Health Sciences Research Committee from the Ministry of Health, Malawi, which ruled that formal approval was not necessary for that type of study. In addition, the National Health Sciences Research Committee as well as the District Health Office of the Ministry of Health ruled that written consent was not necessary for this type of operational research, which should in fact be routine practice in any Malawian district hospital in order to monitor clinical performance. Nevertheless, verbal informed consent was obtained from all included women or their relatives (in case of maternal mortality) for the original study before collecting their information into the database. All results were de-identified and none of the information collected in the database could be traced back to any individual patient. The District Health Office of the Ministry of Health took part in the study design and ensured that the study was performed conform national guidelines. This present study used only the de-identified data contained in the original database. For this present type of study, further informed consent was not required.

### Setting

Thyolo District is an area that had a population of around 600 000 in 2004, with an adult HIV-prevalence of 21% and a total fertility rate of 5.7 [10]. It is located in Southern Malawi, a low-income country in sub-Saharan Africa. Similar to other districts in Malawi and the wider region, the health system is comprised of one large public district hospital and several small peripheral government- and mission-run health facilities. In many districts, non-governmental organizations provide technical and logistic support to the public health system including in Thyolo where Médecins Sans Frontières is present [11]. Care in the public health system is provided free-of-charge.

### Participants

A prospective study of maternal mortality and MNM was performed in Thyolo District Hospital over a two-year period from September 2007 to August 2009 (the ‘4M-Study’: study of maternal mortality and maternal morbidity in Thyolo). Forty-six cases of maternal mortality and 340 women with MNM defined according to disease-specific criteria were identified [7].

The near miss criteria applied in the ‘4M-study’ were: (1) uterine rupture, defined as the occurrence of clinical symptoms (pain, fetal distress, acute loss of contractions, hemorrhage) or intrauterine fetal death that led to laparotomy, at which the diagnosis was confirmed, or laparotomy for uterine rupture after vaginal birth [12]; to this definition we added rupture confirmed by autopsy or clinical symptoms with a high suspicion of rupture in case of death [13]; (2) eclampsia or severe pre-eclampsia with a maternal indication for termination of pregnancy; (3) major obstetric hemorrhage (including hemorrhage from complicated abortions and ectopic pregnancies), defined as a fulfilled need for transfusion of at least two units of 450 ml of whole blood (we adjusted the commonly cited criterion of four units [14] because of scarcity of blood for transfusion in the local setting) or a hemoglobin level below 6 g/dl measured after vaginal bleeding or an estimated blood loss of more than 1 liter; (4) severe obstetric and non-obstetric peripartum infections, defined as all infections for which intravenous antibiotics or intravenous anti-malarials were prescribed or surgical treatment was performed, as well as neoplasms resulting primarily from HIV-infection (e.g. Kaposi's sarcoma and HIV-associated lymphoma); (5) any other complication the clinician considered severe, with the qualification ‘severe’ confirmed by at least two senior clinicians (a small rest group that turned out to comprise only 5% of the total number of MNM cases)[7]. These criteria derived from similar international studies [14]–[16].

In the 340 women who sustained MNM, 375 MNM diagnoses were made: 119 infections, 119 major obstetric hemorrhages, 75 cases of (severe pre-)eclampsia, 43 uterine ruptures and 19 other complications. Case fatality rates ranged from 4% in the (pre-)eclampsia group to 16% in the infection group. HIV-infection played a role, with 30% of MNM cases and 50% of maternal deaths occurring in HIV-positive women. Systematic obstetric audit and feedback took place in the same period, during which a significant reduction of maternal complications was found [7].

### Intervention

We revisited the medical records of women included into the 4M-study [7]. Medical records included the admission file, labor graph, antenatal records and audit reports. Obstacles to correctly complete the WHO-MNM tool were identified by studying inter-assessor variance and calculating the intraclass correlation coefficient for each type of criterion (A, B and C). This was done by having subsets of cases assessed by four assessors (TvdA, JL, TM, JS) who independently completed the MNM tool for each case within the subset. Differences between the assessors were discussed and consensus was reached upon how to apply the criteria given in the tool. Based on this consensus, the WHO-MNM tool was completed for each woman by two of the authors (TvdA and JL). Correct completion was then verified by a third investigator (JB). JS and TM are gynecologists-obstetricians with extensive experience in similar settings, JB and TvdA are residents in obstetrics and gynecology and worked in Thyolo District as general practitioners for several years. JL is a medical student with a special interest in obstetrics in low-resource settings.

### Data collection and analysis

From all completed MNM tools the following parameters were collected into Microsoft Excel: inclusion diagnoses (A-, B- and C-criteria groups and individual diagnoses A0–A4, B0–B3, C0–C6, see Annex A), maternal and perinatal mortality, mode of delivery, and contributory conditions (HIV, anemia, previous caesarean section and obstructed labor). All parameters were analyzed using SPSS Version 19.0 software package: proportions of each parameter were calculated with the significance level set at 5%. The intraclass correlation coefficient for each type of criterion (A, B and C) was assessed by first calculating the intraclass correlation coefficient for each separate criterion (A0–A4, B0–B3, C0–C6).

## Results

General characteristics of the 386 included women are shown in Table 1.

**Table 1:** General characteristics of all women assessed (n = 386).

Characteristic	Specified	N	Percentage of total
Mortality	Maternal	46	11.9
	Perinatal	106	27.5
Mode of assisted delivery	Cesarean section	134	34.7
	Vacuum extraction*	17	4.4
HIV-positive		120	31.1
Obstructed labor		81	21.0
Previous caesarean section		37	9.6

* not indicated in WHO tool

Nineteen cases were assessed during the inter-assessor comparison. Concordance between assessors ranged between two out of three (67%) and eight out of eight (100%) for A-criteria, between one out of five (20%) and five out of five (100%) for B-criteria, and between zero out of three (0%) and two out of three (67%) for C-criteria (Table 2). The intraclass correlation coefficients were calculated at 0.72 for A-criteria, 0.59 for B-criteria and 0.47 for C-criteria.

**Table 2:** Criteria identified by assessors to 19 cases. N/A  =  not assessed.

Case	Assessor 1	Assessor 2	Assessor 3	Assessor 4
1	A0,A3,B0	N/A	N/A	A0,A3,B0,C0,C6
2	A4,B0,B2	N/A	A4, B2	N/A
3	A4,B2	N/A	N/A	B2
4	A2,C5	N/A	A2	N/A
5	B0	N/A	N/A	A3,B0,B2
6	B0, B2	N/A	B0, B2	N/A
7	A3, B0	N/A	N/A	A3, B0, C4
8	A4,B2	N/A	A4, B2, C6	A4, B0, B2
9	B0	N/A	N/A	B0,B2
10	A2,C5	N/A	N/A	A2
11	N/A	A3	N/A	A3,C1
12	N/A	A3,B0	N/A	N/A
13	N/A	A4, B0, B2, C6	A4, B0, B2, C6	N/A
14	N/A	A3	A3	N/A
15	N/A	A0, B0, C3	B0	B0, B2, C3
16	N/A	A3	N/A	A3, B0, C4
17	N/A	A0,A4,B0,B2,C6	A4, B0, B2, C6	N/A
18	N/A	A1	A1	N/A
19	N/A	A1	A1	N/A

The most important difficulties to fill in the tools observed by the assessors are shown in Table 3 and the solutions agreed between assessors in Table 4. Assessors agreed unanimously that all of the cases they assessed constituted ‘maternal near miss’ according to the WHO-definition: ‘a woman who nearly died but survived a complication that occurred during pregnancy, childbirth or within 42 days of termination of pregnancy’.

**Table 3:** Difficulties perceived by assessors.

1.	No A-inclusion criterion for antepartum hemorrhage, despite consensus that all assessed cases of APH constituted MNM.
2.	Not clear whether to include convulsions as part of eclampsia under C5 (neurological dysfunction: uncontrollable fits?).
3.	Ectopic pregnancies and their complications are not part of the disease-specific (‘A’) criteria.
4.	Unclear which infections can be defined as ‘severe systemic infections’ (A3).
5.	Unclear whether to include a suspected ruptured uterus under criterion A4 or not.
6.	Unclear whether to include uterine repair (in order to spare the uterus) and hysterectomy for uterine rupture under C6 (uterine dysfunction).
7.	Not clear whether any caesarean section should be included as laparotomy (B2).
8.	Not clear which definition of shock should be used.
9.	Unclear what is meant by C5 (hepatic dysfunction): only in the presence of pre-eclampsia?
10.	Malaria treatment is not part of the process indicators.
11.	Not clear whether blood transfusion as a process indicator should be based on a minimum of units transfused.

**Table 4:** Agreed solutions.

1.	Do not include APH-cases under ‘A-criteria’.
2.	Do not include convulsions as part of eclampsia under C5, unless they fulfill the criterion ‘uncontrollable fits’ mentioned in the tool: mortality or continued fits despite administration of anticonvulsants.
3.	Do not include ectopic pregnancies unless cases strictly fulfill any other criteria.
4.	Include all cases for which intravenous antibiotics or intravenous anti-malarials or surgical treatment were used.
5.	Include cases of suspected uterine rupture only if they fulfill the definition used in the 4M-study.
6.	Do not include repair for uterine rupture under C6, but do include hysterectomy for uterine rupture, since the criterion is explicitly described as ‘hemorrhage or infection leading to *hysterectomy*’
7.	Do not include caesarean section under B2; only include ‘other’ laparotomies.
8.	Use the definitions provided by Say et al.: shock is ‘a persistent severe hypotension, defined as a systolic blood pressure <90 mmHg for ≥60 minutes with a pulse rate at least 120 despite aggressive fluid replacement (>2l) (6).
9.	Strictly apply definition of hepatic dysfunction as given in MNM tool: only jaundice in presence of pre-eclampsia and severe acute hyperbilirubinemia to be included.
10.	Record cases in which malaria treatment was given separately.
11.	Include all cases in which *any* blood transfusion was given, regardless of the amount.

Of all 386 women that had initially been included into the 4M-study, 341 (88%) fulfilled one or more of the WHO disease-specific A-criteria. The remaining 45 cases, which did not meet any A-criteria, were: 23 antepartum hemorrhages, six ectopic pregnancies, three abortions complicated by severe hemorrhage, two cases of Kaposi's Sarcoma, two cases of stroke, two cases of very severe anemia during pregnancy, two puerperal psychoses, one sudden cardiac arrest, one obstructed labor with necrosis, one burst abdomen post-caesarean, one vaginal tear due to unsafe abortion, and one maternal death with unknown cause.

Of the 386 women, 179 (46%) fulfilled one or more ‘B-criteria’. In total 224 B-events were recorded: 163 cases in which blood transfusion had been given and 61 cases in which laparotomy had been performed. There had been no intensive care admissions or invasive radiological procedures, since neither of these services was available at this facility.

Only 85 women (22%) fulfilled organ failure based C-criteria. In total 90 C-events were recorded. Table 5 shows the number of events recorded in each (sub-) category.

**Table 5:** Inclusions per (sub-) category in 386 women with 421 critical events.

Category	N	Sub-category	N	Percentage of inclusions per category
A: disease	392	0: PPH	107	27%
		1: Pre-eclampsia	20	5%
		2: Eclampsia	71	18%
		3: Infection	148	38%
		4: Ruptured uterus	46	12%
B: intervention	224	0: Blood products	163	73%
		1: Interventional radiology	0	n/a
		2: Laparotomy	61	27%
		3: Admission into ICU	0	n/a
C: organ failure	90	0: Cardiovascular	29	32%
		1: Respiratory	14	16%
		2: Renal	1	1%
		3: Coagulation/Hematologic	4	4%
		4: Hepatic	13	14%
		5: Neurologic	8	9%
		6: Hysterectomy	21	23%

## Discussion

Our findings have several implications for the approach to finding universal criteria for MNM, especially in resource-poor settings. Firstly, the application of disease-, intervention- and organ failure specific criteria would lead to different proportions of severe acute maternal morbidity being included as maternal near-miss. Disease-specific criteria ‘pick up’ most of the severe morbidities, while the organ failure criteria as preferred by WHO would lead to a considerably lower number of ‘near-miss’ cases identified. At district level in a low-resource setting, the absence of sophisticated laboratory diagnostics and the lack of manpower to perform extensive clinical monitoring clearly prevent inclusion of MNM based on C-criteria.

Secondly, the inter-assessor concordance and intraclass correlation show that the fulfillment of C-criteria appears to be subject to perceptual differences between assessors, to a larger extent than the fulfillment of A- or B-criteria. This indicates that the use of C-criteria would have to be accompanied by extensive instructions to health workers as to how to apply these criteria in practice.

Thirdly, the B-criteria appear to be relatively straightforward and would lead to inclusion of a considerable proportion of clinical MNM, but these criteria do not allow for a significant differentiation among cases, as only two out of four interventions (blood transfusion and laparotomy) are commonly available at district level in low-income settings. Moreover, those cases that would undoubtedly be considered MNM on clinical grounds but in which neither of these two interventions are performed would not be included as MNM in the WHO Tool. These B-criteria could be relevant in order to identify the severity of some of the conditions classified as A-criteria.

In a recent study from a tertiary intensive care facility in Brazil, the use of organ-failure criteria enabled the researchers to identify most maternal near misses. Out of 673 admissions into this unit, 194 ‘near misses’ and 18 maternal deaths (MD) were identified, giving a ‘Mortality Index’ (MI = MD/MNM+MD 100%) of 8.5% [17]. In our cohort, this MI, even using disease based criteria, would be (46/340+46 100%) 12%. Using the organ failure criteria, the MI would be (46/90+46*100%) 34%. In other words, even the ‘potentially life threatening conditions’ in rural Malawi according to the WHO definition would be more deadly than the ‘life threatening conditions’ in urban Brazil.

We agree with the WHO working group that organ failure is a logical pre-stage to death and that identifying organ failure would be a logical manner to determine the severity of maternal illness. However, this identification is difficult, particularly in the absence of sophisticated diagnostics. Moreover, our goal should be to *prevent* organ-system dysfunction. One step is then to learn lessons from ‘potentially life-threatening conditions’, rather than ignoring these. Only when the MI or the case fatality rate of ‘potentially life threatening conditions’ has fallen below 1%, a level that was previously identified as an indicator of acceptable obstetric care by the Averting Maternal Death and Disability Program [18], the focus may be shifted to organ-system dysfunction, although assessing this type of dysfunction may remain difficult in a resource-poor setting.

Based on these study findings, we do not agree with the recent statement that ‘an organ-system dysfunction approach remains as the most epidemiologically sound set of criteria’ [19]. We suggest that the disease-specific A-criteria would be the most appropriate MNM criteria in low-resource settings where the MI is higher than 1%. If antepartum hemorrhage and complications of ectopic pregnancy and abortion would be included under these A-criteria, most of the severe acute morbidity would be included as MNM. We felt that the inclusion of approximately 190 women on a yearly basis, or 16 women per month, made for a manageable workload [7]. We also feel that most of the severe morbidity cases based on modified disease-specific criteria would fulfill the definition of MNM given by WHO.

One limitation of our study is that it built on the outcome of a previous study of maternal morbidity and mortality that relied on independent inclusion criteria [7]. These study criteria will account for some underreporting of total maternal morbidity that could be considered MNM. For instance, women who received only one unit for blood transfusion would not have been included into the ‘4M’-study unless major blood loss would have been recorded or a very low hemoglobin would have been measured, and were therefore not included into the present analysis, although they would have fulfilled the WHO B0-criterion. It is likely that, unless the disease-specific criteria for hemorrhage are adjusted to include ‘major’ hemorrhage only, over-representation of hemorrhage as MNM would occur. In addition, our application of the 4M-criterion for the inclusion of severe systemic infection (intravenous medication or surgical treatment) could lead to over-reporting. However, our previous finding that even with this relatively ‘mild’ criterion the case fatality rate for peripartum infections stood at 16% (the highest index of all different morbidities), we do not think that over-reporting played any role of importance [20]. In the study setting, potent intravenous antibiotics are relatively scarce and not as commonly used as in many high-income countries. Therefore, use of this type of medication may be an appropriate indicator for the severity of an infection in low-resource settings.

It must also be noted that the identification of severe maternal morbidity in Thyolo had the specific interest of several staff in the district. Audit of maternal morbidity is considered to be a valuable activity by most health workers in the district [21]. Therefore, the results cannot automatically be expected to be similar in other districts. Moreover, since MSF provided some extra laboratory capacity in Thyolo (creatinine- and bilirubin measurements testing, full blood cell counts) some inclusions, particularly in the C-group, could not happen in districts with less sophisticated readings. In other words, in such districts yet a lower proportion of cases with organ failure would be identified.

Reaching consensus on universal criteria to compare maternal outcome across time and space may be a useful undertaking, provided that these criteria would not underestimate poor maternal outcomes in those areas where these are expected to be highest. We subscribe to the statement made initially by the WHO Working Group that the guiding principle for the development of criteria should be that these are ‘feasible for use in any setting regardless of the development status’. Based on the findings of this study, the WHO-MNM approach to use these organ failure based criteria may not fulfill this principle, although these criteria should be tested in similar settings to determine their usefulness.

## Supporting Information

Figure S1 **WHO Maternal Near Miss Tool.**
(DOCX)
