# Supplementary figures and images for: The WHO Maternal Near Miss Approach: Consequences at Malawian District Level
Source: PLoS One. 2013 Jan 25;8(1):e54805. doi: 10.1371/journal.pone.0054805 (PMC3556078; doi:10.1371/journal.pone.0054805)

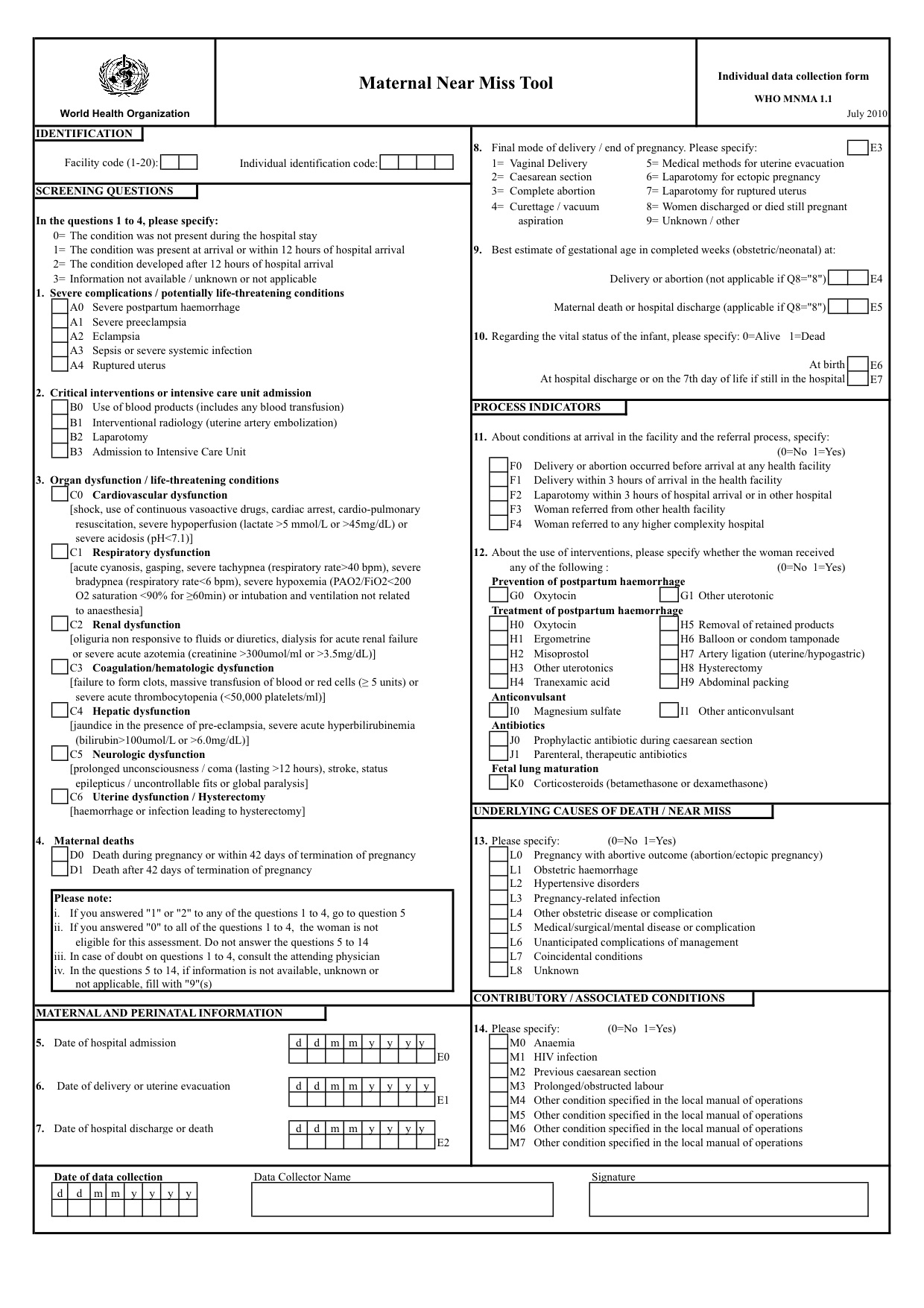

Supplement: Figure S1 — WHO Maternal Near Miss Tool. (DOCX) [file pone.0054805.s001.docx]
